# Hiccough

**Published:** 1891-01

**Authors:** Wm. Steinrauf

**Affiliations:** St. Charles, Mo.


### HICCOUGH.

W. Steinrauf, M. D., St. Charles, Mo.

Mr. D. F——, brother-in-law of an old-school physician in our city, after trying different allopathic measures for a distressing hiccough, came to my office, asking to be relieved. As I was busy with other patients when he came, I had an opportunity to notice the hiccoughing. Within a few moments it occurred four or five times.

Could Homœopathy help him? With the remark: "This will cure you," I dropped ten or fifteen pellets of Nux-vom.$^{dmm}$ on his tongue. The relief was instantaneous! Rejoice and be exceedingly glad, all ye children of men, that Hahnemann lived and made known to the world the beautiful and divine law: *Similia similibus curantur.*

